# A phase I trial of the HIV protease inhibitor nelfinavir in adults with solid tumors

**DOI:** 10.18632/oncotarget.2415

**Published:** 2014-09-06

**Authors:** Gideon M. Blumenthal, Joell J. Gills, Marc S. Ballas, Wendy B. Bernstein, Takefumi Komiya, Roopa Dechowdhury, Betsy Morrow, Hyejeong Root, Guinevere Chun, Cynthia Helsabeck, Seth M. Steinberg, Jaclyn LoPiccolo, Shigeru Kawabata, Erin R. Gardner, William D. Figg, Phillip A. Dennis

**Affiliations:** ^1^ Medical Oncology Branch, National Cancer Institute, Bethesda, MD; ^2^ Biostatistics and Data Management Section, National Cancer Institute, Bethesda, MD; ^3^ Department of Oncology, Johns Hopkins School of Medicine, Baltimore, MD, USA

**Keywords:** Nelfinavir, phase I clinical trial, AKT, endoplasmic reticulum stress, neuroendocrine

## Abstract

Nelfinavir is an HIV protease inhibitor being repurposed as an anti-cancer agent in preclinical models and in small oncology trials, yet the MTD of nelfinavir has not been determined. Therefore, we conducted a Phase Ia study to establish the maximum tolerated dose (MTD) and dose limiting toxicities (DLT) of nelfinavir in subjects with advanced solid tumors. Adults with refractory cancers were given oral nelfinavir twice daily with pharmacokinetic and pharmacodynamic analyses. Twenty-eight subjects were enrolled. Nelfinavir was generally well tolerated. Common adverse events included diarrhea, anemia, and lymphopenia, which were mostly mild. The DLT was rapid-onset neutropenia that was reversible. The MTD was established at 3125 mg twice daily. In an expansion cohort at the MTD, one of 11 (9%) evaluable subjects had a confirmed partial response. This, plus two minor responses, occurred in subjects with neuroendocrine tumors of the midgut or pancreatic origin. Thirty-six percent of subjects had stable disease for more than 6 months. In peripheral blood mononuclear cells, Nelfinavir inhibited AKT and induced markers of ER stress. In summary, nelfinavir is well tolerated in cancer patients at doses 2.5 times the FDA-approved dose for HIV management and showed preliminary activity in tumors of neuroendocrine origin.

## INTRODUCTION

The success of molecularly targeted agents such as imatinib, erlotinib and vemurafenib established the era of personalized medicine in oncology. Yet, for a majority of cancer patients there remains an urgent need for more effective, better-tolerated drugs. The rising price of new oncology drugs [1], and the increasing length and cost of drug development [2], have caused growing concern within the government, academia and pharmaceutical industry. One means of hastening drug development and reducing costs is to identify new indications for already approved drugs, referred to as ‘repositioning’ or ‘repurposing’ [3, 4]. Repurposing established drugs may eliminate the years of pre-clinical pharmacology, toxicology and chemistry normally required for New Molecular Entities, and has successfully delivered drugs previously approved for non-cancer indications such as thalidomide [5, 6] into the clinic, and itraconazole [7] and chloroquine [8] into oncology clinical trials.

We hypothesized that nelfinavir could be repurposed as an anti-cancer agent because of its ability to inhibit the PI3K/Akt/mTOR pathway in preclinical studies. Activation of the PI3K/Akt/mTOR pathway is an important feature in many cancers, and inhibitors of this pathway have been developed as anti-cancer drugs [9, 10]. Inhibition of this pathway can induce toxicities such as dyslipidemia, hyperglycemia, and insulin resistance [11–13], all of which are commonly observed in patients treated with HIV protease inhibitors. This raised the possibility that HIV protease inhibitors might have unappreciated clinical activity as inhibitors of this pathway. Secondly, preclinical studies have shown that nelfinavir has anti-cancer properties. For example, nelfinavir inhibits the PI3K/AKT/mTOR pathway, inhibits cancer cell proliferation, and induces endoplasmic reticulum stress, autophagy and apoptosis [14] at concentrations that have been observed in HIV patients.

Given that a maximum tolerated dose (MTD) of nelfinavir was never established in early HIV clinical trials because dose escalation stopped with suppression of HIV RNA viral load [15, 16], we conducted a Phase Ia, dose escalation study in subjects with advanced solid tumors to determine the MTD and dose limiting toxicities (DLT) of nelfinavir in cancer patients.

## RESULTS

Twenty-eight subjects with a wide variety of tumor histologies and tissues of origin were enrolled between 2007 and 2010 and are included in the analysis (Table 1). The most common cancers were colorectal adenocarcinoma (17.9%), small cell lung cancer (14.3%), non-small cell lung cancer (14.3%), and neuroendocrine tumor of the midgut or pancreas (14.3%). Subjects were heavily pre-treated, with 42.9% having received three or more prior systemic regimens. The majority of subjects (78.5%) had ECOG performance status of 1 or 2.

**Table 1:** Patient Demographics all Treated Patients (n=28)

Demographic	Number	%
**Sex**		
Male	19	67.9
Female	9	32.1
**Age (years)**		
Median	63 (range 24.8–84.8)	
**Race**		
White	24	85.7%
Black	4	14.3%
**Cancer Type**		
Colorectal	5	17.9%
Lung (small cell)	4	14.3%
Lung (non small cell)	4	14.3%
Carcinoid/ NET	4	14.3%
Thyroid	3	10.7%
Renal	2	7.1%
Adenoid Cystic	2	7.1%
Other (sarcoma, head and neck, pancreatic adeno, prostate)	4	14.3%
**Prior Chemotherapy Regimens (range 0–8)**		
0	3	10.7%
1–2	13	46.4%
>3	12	42.9%
**ECOG PS at screening**		
0	6	21.4%
1	16	57.1%
2	6	21.4%

Twenty-four subjects were evaluable for safety, while four subjects withdrew consent for non-toxicity related issues prior to completion of cycle 1 and were replaced. These subjects were considered non-evaluable for the following reasons: one for progression of disease (DL2), two due to patient preference (DL1 and DL4, respectively), and one for protocol deviation (DL4). The numbers of subjects evaluable for toxicity at each dose level were: three each at DL1 (1250 mg bid), DL2 (1875 mg bid), DL3 (2500 mg bid), and DL5 (3750 mg bid), and twelve patients at DL4 (3125 mg bid).

### Safety

**Figure 1:**
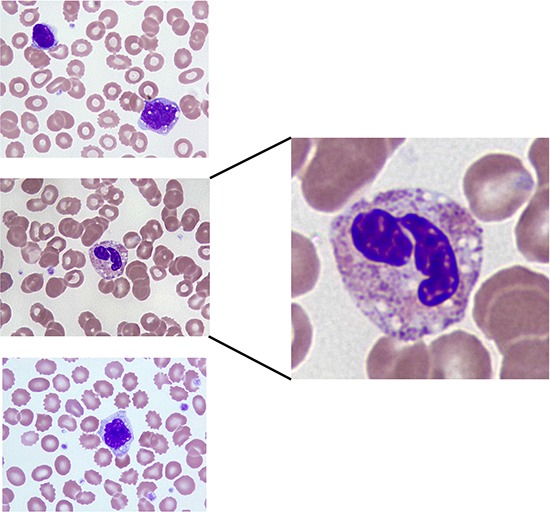
Blood smear showing vacuolated monocytes and neutrophil from a patient that experienced DLT on nelfinavir DL5

Subjects evaluable for toxicity (n=24) received 119 courses at five different dose levels. The median number of cycles was 2 (range 1–35 cycles). No cumulative toxicities were observed. The dose-limiting toxicity was grade 4 neutropenia, which occurred in two of three patients at dose level 5 (Table 2). Nelfinavir-induced neutropenia differed from that observed with conventional cytotoxic chemotherapy in that it was characterized by rapid onset (≤3 days), followed by rapid recovery after discontinuation of drug (≤3 days), and a similar rapid decline with re-challenge. A representative image of a subject's peripheral blood smear that experienced nelfinavir-induced neutropenia (with acanthocytosis) is shown in Figure 1.

**Table 2:** Treatment-related Adverse Events in cycle 1

	DL1 (n=3)		DL2 (n=3)		DL3 (n=3)		DL4 (n=12)		DL5 (n=3)		Total N (%)
	Gr 1-2	Gr 3-4	Gr 1-2	Gr 3-4	Gr 1-2	Gr 3-4	Gr 1-2	Gr 3-4	Gr 1-2	Gr 3-4	
**General**											
Fatigue			**1**				**3**		**1**		**5 (20.8)**
Dehydration							**1**		**1**		**2 (8.3)**
**GI**											
Heartburn							**1**				**1 (4.2)**
Diarrhea			**2**		**1**		**5**	**1**	**3**		**12 (50.0)**
Nausea							**2**				**2 (8.3)**
Bloating/							**2**		**1**		**3 (12.5)**
Anorexia									**1**		**1 (4.2)**
Belching							**1**				**1 (4.2)**
Abdominal pain							**1**				**1 (4.2)**
Flatulence							**1**				**1 (4.2)**
**Metabolic**											
ALT increase			**1**		**1**		**2**		**1**		**5 (20.8)**
Cholesterol increase							**3**				**3 (12.5)**
Hyponatremia							**1**		**1**		**2 (8.3)**
Hyperglycemia					**2**		**2**				**4 (16.7)**
Hypothyroid				**1**							**1 (4.2)**
Hypokalemia							**1**				**1 (4.2)**
Hypophosphatemia									**1**		**1 (4.2)**
Hyperkalemia							**2**		**1**		**3 (12.5)**
Hypoalbuminemia							**3**		**2**		**5 (20.8)**
**Hematologic**											
Anemia							**9**		**1**		**10 (41.7)**
Thrombocytopenia					**1**				**1**		**2 (8.3)**
Leukopenia							**1**	**1**		**2**	**4 (16.7)**
Neutropenia					**1**		**2**			**2**	**5 (20.8)**
Lymphopenia							**6**	**2**	**2**		**10 (41.7)**
**Other**											
Depression							**1**				**1 (4.2)**
Palpitations									**1**		**1 (4.2)**
Insomnia									**1**		**1 (4.2)**

The most common treatment-related adverse events (AE) observed in cycle 1 included diarrhea (50%), anemia (41.7%), lymphopenia (41.7%), fatigue (20.8%), and hypoalbuminemia (20.8%) (Table 2). Rates of AEs attributable to nelfinavir increased with dose escalation, but clinically significant grade 3 or 4 AEs were rare. Grade 3 to 4 AEs of interest include two cases of grade 4 neutropenia at DL5, one case of grade 3 diarrhea at DL4, and a grade 3 hypothyroidism at DL2. The hypothyroid event was due to an interaction between nelfinavir and levothyroxine, which has also been reported in HIV patients taking protease inhibitors and thyroid replacement therapy [17]. Following this event, the protocol was amended to extend the interval between administration of nelfinavir and levothyroxine to at least three hours apart, and no further hypothyroid events were noted in patients taking thyroid replacement therapy.

### Pharmacokinetics

First dose nelfinavir pharmacokinetics were evaluable in 26 subjects across five dose levels, ranging from 1250 to 3750 mg (Supplemental table 1). Non-linear pharmacokinetics were observed following the first dose of oral nelfinavir (Figure 2A). No increase in overall nelfinavir exposure or maximal plasma concentration was seen at doses in excess of 1875 mg. However, variability in exposure was substantially higher at higher dose levels.

**Figure 2A:**
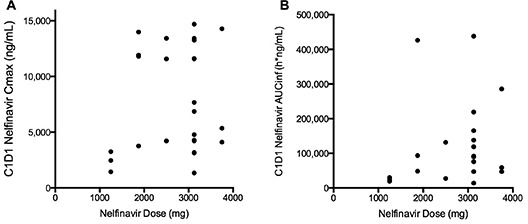
C1D1 Nelfinavir (A) Cmax and (B) AUCinf, by dose Each dot represents an individual patient

Steady state (C2D1) nelfinavir pharmacokinetics were evaluable for 17 subjects (Supplemental Table 2). No significant association between dose and Cmax, Cmin and AUC was observed (p=0.09 to 0.63) (Figure 2B), further exemplifying the non-linear pharmacokinetics of this agent. Nonetheless, there was a trend towards increasing trough concentrations (regardless of morning or evening) with increasing dose.

**Figure 2B:**
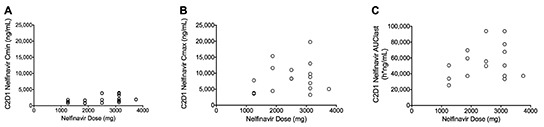
C2D1 Nelfinavir (A) Cmin (immediately prior to dose administration), (B) Cmax and (C) AUClast, by dose Each dot represents an individual patient.

The pharmacokinetics of midazolam, a phenotyping probe for CYP3A4 activity, were evaluated prior to the start of treatment with nelfinavir (C1D-2) and at steady state (C1D20). Complete, paired pharmacokinetic data was evaluable for 19 subjects. A significant decrease in midazolam clearance was observed on day 20, as compared to pre-nelfinavir (p<0.001; Figure 2C). Similarly, a significant increase in exposure and half-life were noted (p<0.05 for each; data not shown).

**Figure 2C:**
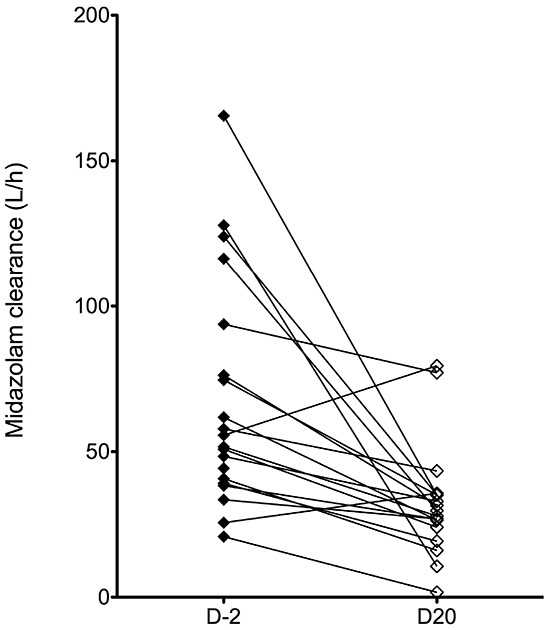
Pairwise comparison of midazolam clearance prior to the start of nelfinavir treatment (D-2) and at nelfinavir steady-state (D20).

### Pharmacodynamics

**Figure 3:**
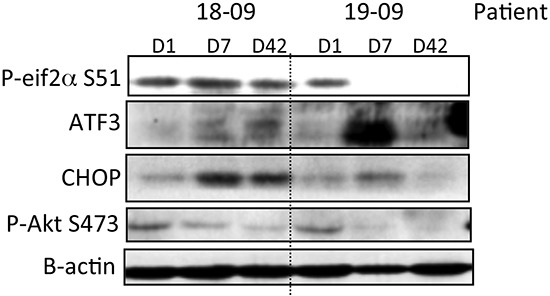
PI3K/AKT inhibition and ER stress pathway induction by nelfinavir in PBMCs of two cancer patients at the MTD

Surrogate biomarkers in PBMCs collected at baseline, D7 and D42 were analyzed by immunoblotting. These biomarkers included evaluation of Akt activation (P-Akt S473) and markers of ER stress (P-EIF2 alpha S51, ATF3 or CHOP). There were no biomarker changes noted in PBMCs collected from subjects at baseline to either DL1 or DL2. Samples from 1 out of 3 subjects in DL3 showed a decrease in P-AKT S473 or an increase in EIF2 alpha S51 phosphorylation. At DL4, 45% of subject PBMCs taken after 7 or 42 days of nelfinavir treatment showed a decrease in P-AKT S473 and the same percentage showed an increase in P-EIF2 alpha S51, indicative of endoplasmic reticulum (ER) stress. In addition, 27% of samples showed an increase in ATF3 and/or CHOP, also suggestive of ER stress. Within DL4, 3 subjects showed changes in 3 of 4 biomarkers evaluated, two examples of which are shown (Figure 3). The rest of the PBMC samples from DL4 subjects showed either change in a single biomarker, or no change from baseline in the 4 biomarkers evaluated.

### Antitumor Activity

**Figure 4:**
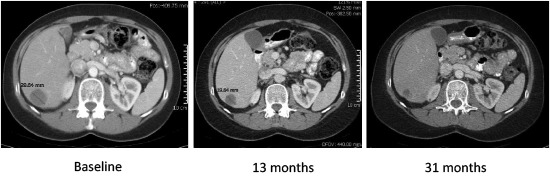
Serial CT images from a subject who achieved a sustained PR response on nelfinavir in target lesion in liver

Twenty-one subjects had an on-study tumor assessment and thus were evaluable for response, eleven in the expansion cohort at the 3125 mg bid MTD, and ten at all other dose levels (Table 3). A patient with progressive neuroendocrine tumor (NET) of the midgut and carcinoid syndrome had a partial response at the MTD (subject 28, Table 3), yielding a 9.1% ORR at the MTD and a 4.8% ORR in the overall population. Minor responses (defined as tumor regressions < 30%) were observed in three additional subjects, all of whom were treated at the MTD. Two of these patients had NET of the midgut or pancreas, (subjects 18 and 26) and one had small cell lung cancer (subject 24, Table 3) although this response was potentially confounded by radiation to the target lesion completed months prior to enrollment. Four subjects treated at the 3125 mg BID MTD (36%) who had progressed prior to study entry, remained on study for 8 months or more. A subject with adenoid cystic carcinoma was on study with stable disease for 11 months. Two of the subjects with NET of the midgut were on study for at least 8 months, and the subject that achieved a PR remained on study for 23.5 months and received nelfinavir off study for an additional six months with stable disease, prior to disease progression in the liver and pelvis (Figure 4). A subject with MEN1 syndrome and pancreatic NET was on study for 12.4 months. In the responders with NET that had carcinoid syndrome with flushing and diarrhea, nelfinavir improved these symptoms within 7 days, which was associated with transient decreases in levels of circulating chromogranin, synaptophysin, and/or urinary 5-HIAA.

**Table 3:** Evaluable subject response and duration of therapy on Nelfinavir

Subject	Age	Diagnosis	Dose Level	Best Response	Duration of therapy (mos.)
1	62	SCLC	1250 mg BID	PD	1.4
3	63	SCLC		PD	1.4
4	77	SCLC		PD	1.4
5	63	Anaplastic Thyroid	1875 mg BID	PD	1.4
7	62	Differentiated Thyroid		PD	2.8
8	65	NSCLC		SD	3.5
9	24	Colorectal	2500 mg BID	PD	1.4
10	75	Pancreatic Adenocarcinoma		PD	2.8
11	71	NSCLC		SD	4.1
12	59	Differentiated thyroid	3125 mg BID	PD	2.1
14	48	Renal Cell Cancer		PD	1.4
17	64	Adenoid Cystic		SD	11.0
18	60	Neuroendocrine tumor		MR	8.3
19	62	Colorectal		SD	4.1
23	64	Prostate		PD	1.0
24	61	SCLC		MR	2.8
25	46	Colorectal		SD	2.8
26	65	Neuroendocrine tumor		MR	12.4
27	67	Neuroendocrine tumor		SD	3.5
28	45	Neuroendocrine tumor		PR	23.5
21	49	Adenoid Cystic	3750 mg BID	PD	1.4

SCLC= small cell lung cancer; NSCLC = non small cell lung cancer; BID = twice daily; PD = progressive disease; SD = stable disease; MR= minor response; PR = partial response

## DISCUSSION

Nelfinavir has pleiotropic mechanisms in cancer cells including induction of ER stress, apoptosis and autophagy [14], inhibition of angiogenesis [18], proteasome activity [19], AKT [14], HIF1alpha [20], site-2 protease [21], and hsp90 [22], as well as radiation sensitization [23]. The fact that the crucial mechanism(s) *in vivo* are not known, combined with the lack of knowledge regarding MTD, informed our decision to perform a dose escalation phase I trial. This study establishes the maximal tolerated dose of nelfinavir in patients with mixed-cancer subtypes at 3125 mg BID, and is the first to describe potential benefit in patients with neuroendocrine tumors. Nelfinavir was well tolerated. At the 3125 mg BID MTD dose, patients did not experience adverse reactions typically associated with either cytotoxic chemotherapy, tyrosine kinase inhibitors, or the Akt/mTOR pathway (such as rash, mucositis and hyperglycemia), nor did they experience significant diarrhea as has been reported with nelfinavir in HIV patients. These observations are consistent with the results of a Phase I trial of nelfinavir in liposarcoma patients where the MTD was not reached; however, auto-induction was noted at the highest dose evaluated (4125 mg BID), and a Phase II trial using a 3,000 mg BID dose is planned [24].

The dose limiting toxicity of nelfinavir is neutropenia, and a blood smear from a patient that experienced this DLT showed the presence of cytoplasmic vacuoles in their neutrophils and monocytes, as well as acanthocytosis. The presence of cytoplasmic vacuoles is similar in appearance to the vacuoles observed in cancer cell lines treated with nelfinavir *in vitro* [14], and the red blood cell morphology is similar that observed in GADD34 knockout mice [25] that have increased levels of ER stress, because GADD34 dephosphorylates eif2alpha [26] and thereby reverses translation inhibition under stress conditions. Cytosolic vacuolization, plus the fact the neutropenia was rapidly reversible, suggests this toxicity may be due to induction of ER stress in mature cellular lineages rather than affecting bone marrow precursor cells. Unexpectedly, several of the common toxicities of protease inhibitors observed in HIV patients, such as central lipodystrophy, dyslipidemia, or hyperglycemia were not frequently observed in our study population.

One patient with NET of the midgut experienced a partial response and two other patients with NET had minor responses and prolonged stable disease after documented progression prior to enrollment. Recently, a trial with the mTOR inhibitor everolimus demonstrated efficacy in pancreatic NET, driven mainly by stable disease [27]. It is not known whether nelfinavir decreases tumor growth in neuroendocrine tumors by inhibition of the PI3K/AKT/mTOR pathway, which can be activated in pancreatic neuroendocrine tumors by mutations in the mTOR pathway [28], or by causing cytotoxicity due to ER dysfunction and protein misfolding in a secretory tumor with high rates of protein synthesis, bioactive hormone release, and turnover.

The MTD of nelfinavir as a single agent in solid tumors is 3125 mg bid, which is 2.5 fold over the doses typically used in HIV patients. The PK data showed potential non-linearity above doses of 1875 to 2500 mg. These data correspond with nelfinavir PK in liposarcoma patients where the authors found non-linearity above 3000 mg bid [24], and suggests that dose escalation of nelfinavir to the 3125 mg bid MTD in future single agent or combination studies in cancer may not be warranted. Although our results and the results in liposarcoma suggest that nelfinavir has activity as a single agent, nelfinavir may have additional efficacy when combined with different types of agents. For example, nelfinavir has been combined with bortezomib in preclinical studies to exploit proteotoxicity as a mechanism of cancer cell death [29]. Nelfinavir can also be safely combined with other chemotherapies as well as radiation, because Phase I trials that used the FDA-approved dose of nelfinavir with concurrent chemoradiation in pancreatic cancer [30], rectal [31] and NSCLC [32] have been reported.

In conclusion, nelfinavir has great promise for repositioning as an anti-cancer agent, as its DLT is readily reversible, is otherwise well tolerated, and has preliminary signs of anti-tumor activity, especially in patients with NET of midgut and pancreatic origin. Future trials are planned.

## METHODS

### Ethics

The National Cancer Institute (NCI) intramural Institutional Review Board (IRB) approved the study prior to its initiation. Subjects provided written informed consent for their participation.

### Study Design

This single institution, open-label, Phase Ia dose escalation study was performed to establish the MTD and DLT of nelfinavir in subjects with advanced refractory solid tumors. The study used a modified Fibonacci scheme, in which cohorts of three to six subjects were entered at each dose level until two patients developed DLT. If two or more subjects encountered DLT, then the MTD was exceeded. The MTD was defined as the dose level at which less than 2 of 6 subjects experienced DLT. Each cycle lasted 21 days. Up to 6 additional patients were allowed to be enrolled at the MTD in order to better establish potential rates of toxicity.

### Subject Selection

Subjects needed to have histologically confirmed advanced cancer that had relapsed following or progressed through standard therapy, Eastern Cooperative Oncology Group (ECOG) performance status 0 to 2; expected survival of at least 3 months, adequate hematologic and renal function; total bilirubin less than the upper limit of normal (ULN) and serum AST and ALT < 2.5 × ULN, stable treated brain metastases, no uncontrolled inter-current illness, no strong CYP3A4 inhibitors or inducers, no escalating doses of corticosteroids, no pregnant or lactating women, and no chemotherapy or radiation within 28 days.

### Clinical Care of Patients

Physical examinations, toxicity assessments and laboratory analyses were conducted weekly during cycle 1 and then every three weeks beginning cycle 2. An electrocardiogram was performed every cycle, and restaging scans were performed at baseline and every two cycles thereafter. Subjects remained on study until disease progression (as determined by RECIST), severe toxicity, or individual choice.

### Drug Administration

Nelfinavir was supplied by the Clinical Pharmacy at the NIH Warren Grant Magnuson Clinical Center (Bethesda, MD). Nelfinavir mesylate 625 mg tablets were dispensed.

### Subject Accrual

The starting dose of nelfinavir was the FDA-approved dose in HIV patients of 1250 mg twice daily, which comprised dose level (DL) 1. Dose escalation proceeded in 625 mg, twice-daily increments (DL2: 1875 mg, DL3: 2500 mg, DL4: 3125 mg, DL5: 3750 mg, DL6: 4375 mg).

### DLTs

Toxicity was graded according to the National Cancer Institute Common Toxicity Criteria, version 3.0. DLT was defined as any drug-related (possible, probable, definite) grade 4 neutropenia, grade 3 or 4 neutropenia accompanied by fever 100.4 or greater, grade 3 hypercholesterolemia, grade 3 diarrhea, or any other grade 4 adverse event.

### Pharmacokinetic Methods

To determine CYP3A4 phenotype, a single 3 mg oral dose of midazolam hydrochloride, a CYP3A4 substrate, was administered at day -2 (2 days prior to commencing nelfinavir) and again at steady state (cycle 1 day 20). Serial venous blood samples were collected at 15, 30, 60, 120, 180, 240, and 300 minutes following administration of midazolam. Nelfinavir pharmacokinetics (PK) were obtained after single dose administration, cycle 1 day 1, and again at steady state, cycle 2 day 1. Serial venous blood samples were collected pre-dose and at 1, 2, 3, 4, 5, 6 and 12 hours after administration. Plasma concentrations of midazolam and nelfinavir were determined with a validated assay employing high performance liquid chromatography (HPLC) with mass spectrometric detection. Noncompartmental pharmacokinetic analyses were performed with WinNonlin 5.2 (Pharsight). Individual concentration-time profiles were constructed for each patient, course and drug resulting in four profiles per patient (two each for midazolam and nelfinavir). The maximum plasma concentration (Cmax) and the time of maximum plasma concentration (Tmax) were the observed values. Drug exposure was estimated using the area under the concentration-time curve (AUC). The area under the concentration-time curve (AUC) from time zero to the time of the final quantifiable sample (AUClast) was calculated using the linear trapezoidal method. The area under the concentration-time curve from time zero to infinity (AUC_inf_) was calculated by extrapolation, using the terminal rate constant from the last measurable concentration.

### Pharmacodynamic Analysis

Peripheral blood mononuclear cells (PBMCs) were collected at baseline, and then twice after starting nelfinavir: cycle 1 day 7 and cycle 1 day 42. Levels of P-AKT S473, P-EIF2 alpha S51, ATF3 and CHOP in PBMCs before and after nelfinavir were analyzed by immunoblotting. Blood was collected in Vacutainer CPT collection tubes with sodium heparin (BD Biosciences cat# 362753) and mononuclear cells were isolated as per the manufacturer's instructions. PBMCs were washed with PBS and lysed in 2X LSB buffer [33], aliquotted and stored at -80°C. For analysis, equal amounts of protein per sample were run on 10-12% SDS-PAGE gels, then transferred onto nitrocellulose membranes. Membranes were blocked in 5% milk in TBS + 0.1% tween (TBST) and incubated with primary antibody diluted in 5% BSA in TBST at 4 degrees overnight. The next day membranes were washed, incubated with HRP-linked secondary antibody and developed using ECL chemiluminescent reagent (GE Healthcare) and X-ray film as per the manufacturers instructions. The amount of P-AKT S473, P-EIF2 alpha S51, ATF3 or CHOP in the PBMCs at C1D7 and C1D42 were compared against a baseline sample for each patient, (prior to receiving nelfinavir). Antibodies against P-AKT S473 #4060, P-EIF2 alpha S51 #3398 and β-Actin #4970 were from Cell Signaling Technology (Danvers, MA). Antibodies to CHOP sc-7351 and ATF3 sc-188 were from Santa Cruz Biotechnology (Santa Cruz, CA).

### Tumor Evaluation

Tumor response was assessed according to Response Evaluation Criteria in Solid Tumors (RECIST) [34] every two cycles.

### Statistical Methods

Associations between pharmacokinetic parameters and dose level of nelfinavir were determined using an exact Jonckheere-Terpstra test for trend [35]. Comparison of pharmacokinetic parameters between two time points on paired subjects was performed with a Wilcoxon signed rank test. All p-values are two-tailed.

## SUPPLEMENTARY TABLES


