# Supplementary material for: A phase I trial of the HIV protease inhibitor nelfinavir in adults with solid tumors
Source: Oncotarget. 2014 Sep 6;5(18):8161–72. doi: 10.18632/oncotarget.2415 (PMC4226674; doi:10.18632/oncotarget.2415)
Supplement: Supplementary file 1 [file oncotarget-05-8161-s001.pdf]

SUPPLEMENTARY TABLES

Supplementary Table 1: Cycle 1 Day 1 Nelfinavir Pharmacokinetics

	Tmax (h)			Cmax (ng/mL)			AUClast (h*ng/mL)			AUCinf (h*ng/mL)			CL_F (mL/h)			T1/2 (h)		
**Dose (mg)**	*Mean*	*SD*	*n*	*Mean*	*SD*	*n*	*Mean*	*SD*	*n*	*Mean*	*SD*	*n*	*Mean*	*SD*	*n*	*Mean*	*SD*	*n*
**1250**	3.7	0.6	3	2,387	910	3	16,621	5,554	3	24,502	5,394	3	52,714	11,665	3	6.6	1.6	3
**1875**	3.8	2.1	4	10,370	4,521	4	73,884	33,445	4	189,424	206,340	3	21,034	17,145	3	11.9	8.3	3
**2500**	6.0	5.2	3	9,738	4,870	3	61,035	31,972	3	79,443	73,789	2	55,342	51,404	2	4.5	3.2	2
**3125**	4.8	1.0	13	7,639	4,544	13	56,853	32,532	13	139,773	120,008	10	49,020	63,890	10	9.2	5.7	10
**3750**	2.7	0.6	3	7,918	5,551	3	61,520	44,096	3	130,571	134,507	3	52,114	34,636	3	9.2	5.2	3

Supplementary Table 2: Cycle 2 Day 1 Nelfinavir Steady State Pharmacokinetics

	Tmax (h)			Cmax (ng/mL)			Morning Trough (ng/mL)			Evening Trough (ng/mL)			AUClast (h*ng/mL)			CL_F (mL/h)		
**Dose (mg)**	*Mean*	*SD*	*n*	*Mean*	*SD*	*n*	*Mean*	*SD*	*n*	*Mean*	*SD*	*n*	*Mean*	*SD*	*n*	*Mean*	*SD*	*n*
**1250**	4.4	1.2	3	5068	2347	3	2221	1435	3	1720	781	3	36,659	12,791	35%	36,636	11,821	3
**1875**	3.7	1.5	3	10492	5534	3	1989	2105	3	1946	899	3	55,481	16,587	30%	35,588	12,426	3
**2500**	4.0	1.7	3	9221	1566	3	2868	1751	3	3649	3579	3	66,480	23,923	36%	40,343	12,107	3
**3125**	3.3	1.4	7	9552	5507	7	4597	3222	6	2883	1347	6	60,015	23,722	40%	59,826	25,144	6
**3750**	1.0	-	1	5058	-	1	3160	-	1	1057	-	1	37,309	-	-	98,207	-	1
